# Discrepancy between Knowledge and Perceptions of Dietary Omega-3 Fatty Acid Intake Compared with the Omega-3 Index

**DOI:** 10.3390/nu9090930

**Published:** 2017-08-24

**Authors:** Sowmyanarayanan V. Thuppal, Clemens von Schacky, William S. Harris, Katherine D. Sherif, Nigel Denby, Suzanne R. Steinbaum, Bryan Haycock, Regan L. Bailey

**Affiliations:** 1 Department of Nutrition Science, Purdue University, Room 143, 700 West State Street, West Lafayette, IN 47906, USA; tvsowmy@gmail.com; 2 Department of Preventive Cardiology, Medizinische Klinik and Poliklinik I, Campus Innenstadt, Ludwig Maximilians University, Ziemssen str.1, D-80336 Munich, Germany; c.vonschacky@omegametrix.eu; 3 Department of Medicine, Sanford School of Medicine, University of South Dakota, Health Science Center, 1400 West 22nd Street, Sioux Falls, SD 57105, USA; Bill@omegaquant.com; 4 OmegaQuant Analytics, LLC, 5009 W. 12th Street, Suite 8, Sioux Falls, SD 57106, USA; 5 Jefferson Women’s Primary, 211 S. 9th Street, Walnut Towers, Ste. 401, Philadelphia, PA 19107, USA; Katherine.Sherif@jefferson.edu; 6 Grub4Life and People Matter TV, 40 Bowling Green Lane, Clerkenwell, London EC1R 0NE, UK; nigel@buddypower.net; 7 Women and Heart Disease Center, Lenox Hill Hospital, 110 East 59th Street, New York, NY 10022, USA; drsrsteinbaum@gmail.com; 8 Department of Nutrition and Integrative Physiology, University of Utah, 201 South Presidents Circle Room 201, Salt Lake City, UT 84112, USA; bryan.haycock@utah.edu; 9 Department of Nutrition Science, Purdue University, Stone Hall, Room 143A, 700 West State Street, West Lafayette, IN 47906, USA

**Keywords:** Biomarker, dietary perception, Omega-3 index, fatty acids

## Abstract

Little is known about the relationship between perceptions of nutrient adequacy and biomarkers of nutrition status. This cross-sectional study of U.S. and German adults (*n* = 200; 18–80 years) compared dietary practices, knowledge, and beliefs of omega-3 fatty acids (O3-FA) with the omega-3 index (O3-I), an erythrocyte-based biomarker associated with cardiovascular disease (CVD) risk. More than half of adults believed that O3-FAs are beneficial for heart and brain health and could correctly identify the food sources of O3-FA. However, the mean O3-I in the U.S. (4.3%) and Germany (5.5%) puts the majority of adults sampled (99%) in intermediate or high CVD-risk categories. More Americans were considered at high CVD-risk (40%) when compared with Germans (10%). In the U.S., but not Germany, women had a significantly higher O3-I than men (4.8% vs. 3.8%, *p* < 0.001). In the intermediate CVD-risk group, about one-third of adults in both countries (30% in the U.S. and 27% in Germany) believed their diet was adequate in O3-FA. Notably, mean O3-I concentrations did not significantly differ with dietary perceptions of adequacy. More adults in Germany (26%) than in the U.S. (10%) believed that dietary supplements are needed to achieve a balanced diet. In spite of adequate knowledge about food sources and a consistent belief that O3-FA are important for health, very few participants had O3-I concentrations in the range for CVD protection.

## 1. Introduction

The Academy of Nutrition and Dietetics recommends adopting a balanced diet with a variety of nutrients for achieving optimal dietary intakes [1]. Unfortunately, not all nutrients are ubiquitous in the food supply. Fortified foods and dietary supplements help people increase their nutrient intakes [2,3,4,5,6]. Without these strategies, many people do not achieve the Dietary Reference Intakes (DRI) recommendations [2,3,4,5,6]. Omega-3 fatty acids (O3-FAs) are one of the critical nutrients known to be under-consumed, creating a public health concern in both the U.S. and Germany [7,8]. Globally, many adults have an Omega-3 Index below the recommendations for cardiovascular protection [9].

Seafood (both fish and shellfish) and dietary supplements are important sources of O3-FA [10]. The 2015–2020 Dietary Guidelines for Americans recommends consuming at least eight ounces of fatty fish per week [10]. The 2012 German Nutrition Society Report recommends eating 80 g (three ounces) to 150 g (five ounces) of low-fat saltwater fish or 70 g (2.5 ounces) of high-fat saltwater fish per week [11]. However, the current dietary intake of seafood is very low across all age and sex groups in both countries [8,10]. Without adequate fish consumption, it may be difficult to obtain optimal O3-FAs from foods alone, in which case individuals may require dietary supplements or food fortification to achieve recommended intake [8,12].

Previously, a study on perceptions of healthy diet among adults showed that more individuals in the U.S. than Germany reported the use of O3-FA supplements (45% vs. 24%) [13]. Most (80%) adults in both countries believed that O3-FA plays a significant role in overall health [13], but about half of adults believed that their diet was inadequate in O3-FAs. However, very little is known about the relationship between perceptions of dietary intakes relative to biomarkers of O3-FA status. Therefore, the purpose of this study was to compare the perceptions of adequacy and importance of O3-FAs in the diet with O3-FA status measured by the omega-3 index (O3-I) in red blood cells (RBCs).

## 2. Materials and Methods 

### 2.1. Study Design, Participants, and Setting

A cross-sectional survey of adult participants from the U.S. and Germany was conducted between April and May 2016. In the U.S., the study was conducted at Purdue University through the Indiana Clinical and Translational Science Institute and the Department of Nutrition Science. In Germany, the study was directed by the Department of Preventive cardiology, Ludwig Maximilians-Universität München, Munich, Germany and conducted in Munich, Jena, Meerbusch, Hamburg, and Hannover. The study protocol was approved by Institutional Review Boards at both universities. In order to have equal sex representation, a block recruitment method was used. Non-pregnant, non-lactating adults, between 18–80 years, were eligible to participate. The exclusion criteria included the current use of O3-FA dietary supplements and participants with any acute health conditions in the last three months, including myocardial infarction, stroke, and embolism.

### 2.2. Subject Recruitment

Posters explaining the study procedure and eligibility along with the contact details of the study coordinator were posted in and around the study centers. All interested participants were encouraged to call the study coordinators at each site to schedule a visit. At the study visit, information about the study was provided in detail, study eligibility was assessed, and any queries regarding the study procedure were clarified before obtaining the consent to participate in the study.

### 2.3. Study Instruments and Biochemical Measures

Demographic data and diet perceptions were assessed via a questionnaire consisting of close-ended questions with dichotomous, ordinal, and Likert scale response options. Baseline demographic details included age, gender, race/ethnicity, socioeconomic parameters, alcohol and tobacco use, and physical activity. Questions regarding overall health, diet quality, and balanced diet (i.e., diet with all the recommended daily intake of nutrients) were modified after a previous study [13]. Specific questions to understand perceptions about food that contains O3-FA, deficiency diseases due to the lack of the nutrient, adequacy in the diet, intake of supplements, and importance of O3-FAs for overall health were included in the survey. Height and weight were measured using calibrated instruments and body mass index was calculated using the standard formula weight (kg)/height (m)^2^.

Whole blood was collected using an ethylenediamine tetraacetic acid (EDTA) tube for testing for O3-I. Samples were centrifuged at 3000 rpm at room temperature for 15 minutes. Plasma and buffy coat were removed and the RBC pellets were stored at −80 °C. In the U.S., all samples were shipped to OmegaQuant Analytics (Sioux Falls, SD, USA), and in Germany all samples were sent to Omegametrix (Martinsried, Germany) on dry ice for testing for O3-I. Omega-3 index is a biomarker for O3-FA, which is calculated as a sum of eicosapentaenoic acid (EPA) and docosahexaenoic acid (DHA) content in the RBC membranes and reflects the long-term intake of EPA and DHA [14,15,16]. An O3-I of ≥8% is the recommended optimal level (i.e., low-risk) range, 4–8% is considered an intermediate risk for cardiovascular disease (CVD), and O3-I of ≤4% is considered as high risk for CVD [15].

### 2.4. Data Analysis

Statistical analysis was performed using SAS statistical software 9.4 (SAS Institute Inc., Cary, NC, USA). Means and proportions were computed to describe the baseline characteristics of the individuals enrolled in the study. To compare the differences between sex and O3-I risk categories, an ANOVA (Analysis of Variance) was used for continuous variables with normal distributions, non-parametric tests for continuous variables with non-normal distributions, and chi-square test for categorical variables (*p*-value < 0.05).

## 3. Results

### 3.1. Participants 

Participants in Germany (mean: 40 years; range 18–80 years) were, on average, older than participants in the U.S. (mean: 29 years; range 18–71 years) (Table 1). Most participants had a high school education or higher and had annual incomes <$50,000USD annually (data not shown). All participants that had reported performing some kind of physical activity during the last seven days, and the mean BMI was in the healthy range (18–25 kg/m^2^) in both countries. Men had a higher BMI than women in both countries but no other sex differences were noted within each country. The majority of adults in both countries reported alcohol use and more participants in Germany were current smokers than in the U.S. Most adults rated improving or maintaining health as either important or very important (Supplemental Table S1).

### 3.2. Perceptions of Diet

Almost all the participants (99%) reported that consuming a balanced diet is important, but only 50% from the U.S. and 41% from Germany thought that the food that they eat is nutritionally balanced (data not shown). More than half of the participants in the U.S. (69%) and in Germany (56%) believed that a balanced diet can be achieved through food alone and that dietary supplements are not required (data not shown). More adults in Germany (26%) than in the U.S. (10%) believe that dietary supplements are needed to achieve a balanced diet (data not shown).

**Figure 1:**
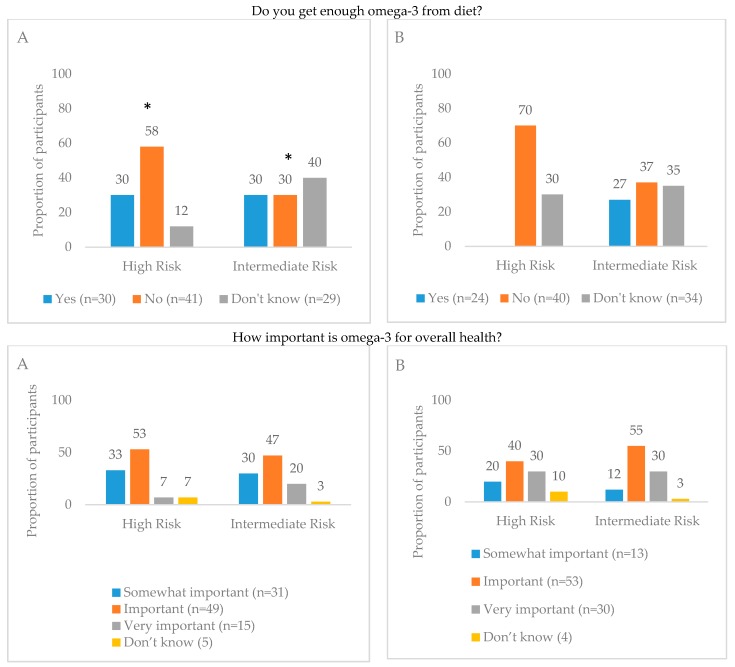

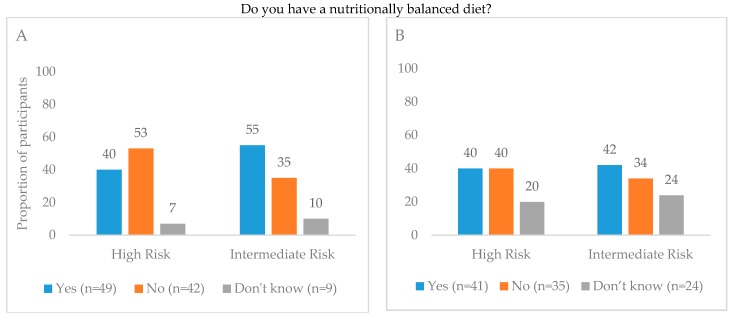
Comparison of perceptions of omega-3 fatty acids in the diet and their importance to health and omega-3 index in the United States (Panel **A**) and Germany (Panel **B**). High Risk—Omega-3 index ≤ 4% (*n* = U.S., *n* = 40; Germany, *n* = 10); Intermediate Risk—Omega-3 index 4–8% (*n* = U.S., *n* = 60; Germany = 90); * Statistically significant, *p*-value < 0.05.

All participants believed that O3-FAs are important for health. Both in the U.S. and Germany, O3-FAs were considered important for improving heart health (66% and 61%, respectively), overall wellness (63% and 46%, respectively), and brain health (53% and 57%, respectively). Almost all participants from the U.S. and Germany thought that oily fish (e.g., salmon, tuna, and sardines) contain O3-FAs (98% and 90%, respectively) (Supplemental Figure S1). However, only 30% of individuals in the U.S. and 24% of individuals in Germany believed that they consume sufficient amounts of O3-FAs from their diet. Regardless of O3-I and country, about one-third of adults are not sure if their diet is balanced or if they get enough O3-FA from their diet (Figure 1).

### 3.3. Biomarker of O3 Status

**Figure 2:**
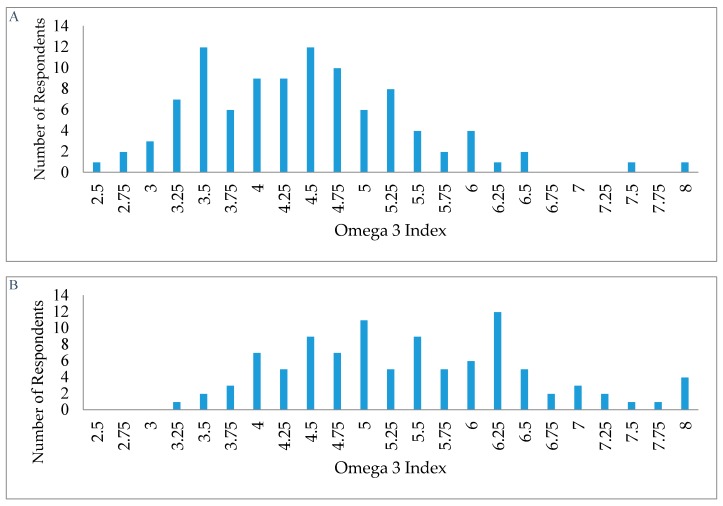
Individual Omega-3 fatty acid values in the United States (Panel **A**) and Germany (Panel **B**).

The mean O3-I values in the U.S. and Germany were 4.3% and 5.5%, respectively (Table 2). None of the study participants from the U.S. and only four individuals and Germany had an O3-I in the recommended optimal range. The distribution of O3-I values are shown in Figure 2. We merged the ‘optimal range’ category and ‘intermediate risk for CVD’ category together for German participants as sample size did not permit analysis of the optimal group (*n* = 4). Only 10% of participants in Germany were classified as high risk for CVD compared with 40% of U.S. participants (data not shown). Women had a significantly higher O3-I than men in the U.S., while no such sex difference was observed in Germany.

### 3.4. Comparison of Dietary Perceptions and Biomarker Status

O3-I was not significantly different between people who perceived their diet as adequate in O3-FA and those who did not perceive their diet as adequate in both study sites (Table 2). While comparing the dietary perceptions and O3-I (Figure 1), around 30% of participants in both risk categories from the U.S. believed that they receive enough O3-FAs from their diet. In Germany, 27% of individuals in the intermediate risk category believed that they received enough O3-FAs from their diet compared to none in the high risk category. More participants in the O3-I high risk categories from both the U.S. (53%) and Germany (40%) reported that they perceived their diet as not nutritionally balanced compared to those in the intermediate risk categories. More individuals from the U.S. in the high risk category (58%) thought that they do not get an adequate amount of O3-FAs from their diet compared to the intermediate risk group (30%), while no such differences were seen in Germany. About 16% of adults thought that O3-FA deficiency existed in the U.S., compared to 70% in Germany.

## 4. Discussion

Almost all individuals in our study, regardless of country, had a fair knowledge of the health benefits of O3-FAs and correctly identified the food sources of O3-FAs. All participants believed that a balanced diet is important for health; many acknowledged that a balanced diet can be achieved through food alone, with no need for dietary supplements. However, the O3-I biomarker status was below the optimal range in almost all individuals from both countries. This discrepancy may be due to a misconstrued interpretation of perceptions of what constitutes a nutritionally balanced diet or other factors that may prevent them from achieving the intakes for optimal status.

In spite of having similar knowledge and perceptions about O3-FA, the mean O3-I was higher and the number of individuals classified as high risk for CVD was lower in Germany compared to the U.S. One possible reason for this discrepancy could be a greater consumption of fish products in Germany compared to the U.S. Secondly, even when consumed, not all fish contain sufficient amounts of EPA and DHA. Studies in the U.S. have shown that the concentration of EPA and DHA has decreased in farm grown fish across time [17]. Consumption of EPA and DHA fortified foods may have also increased in both countries, but little data are available to make cross country comparisons [18,19,20].

Seafood is a major source of EPA and DHA, and it has been well established that maintaining higher blood concentrations of O3-FA is associated with reduced risk of CVD [21,22,23,24,25,26,27]. The 2015–2020 Dietary Guidelines for Americans [8] and the 2012 German Nutrition Society Report [11] provides country-specific guidelines for fish consumption to maintain optimal concentrations of O3-FAs. In spite of these recommendations, studies have shown that seafood consumption is well below recommended levels in both U.S. and Germany [8,9,28]. Our data suggests that none of the individuals from the U.S. and only four individuals from Germany had blood values in the optimal O3-I range for CVD protection. We did not include dietary supplement users in this study so the majority of adults in both countries are likely not complying with the dietary recommendations for fish intake [8,10]. Nutrition knowledge has a positive impact on the quality of diet [29,30,31], but other socioeconomic factors, including education, income, sex, and accessibility to food, also play important roles in improving the quality of food intake [32,33,34,35]. A fear of seafood contamination with mercury may similarly limit its intake.

The O3-I has been demonstrated to have a good dose-response relationship with dietary intakes of O3-FAs, and the O3-I has been a validated surrogate biomarker of dietary intakes [21,36,37,38]. Omega-3 status has been related to a wide range of health outcomes. O3-FAs may be beneficial for predicting survival outcomes in coronary heart disease [15], insulin sensitivity [39], for improving bone health [40], for treatment of depression [41,42,43], and for improving cognition and memory [44,45,46,47]. O3-FAs may play an important role in pregnancy for fetal brain development [48,49,50] and may reduce the risk of pediatric allergies [51]. Given the widespread health benefits associated with optimal omega-3 status, public health interventions are required at the national level to identify strategies that will help increase the consumption of foods rich in O3-FAs.

Some limitations of the current study exist, and our findings should be interpreted with these caveats in mind. First, our participants were recruited through a convenience sampling procedure and were relatively young and well educated. Due to small sample size, four individuals from Germany with O3-FA values in the ‘optimal range’ were included in the ‘intermediate risk for CVD category’. In the questionnaire, we defined a balanced diet as a ‘diet with all the recommended daily intake of nutrients’, however there is no general scientific definition for a balanced diet and it can be interpreted differently. Nevertheless, almost all participants in both countries felt consuming a balanced diet was important (99%).

## 5. Conclusions

In spite of adequate knowledge of nutrients and their health effects, individuals did not have adequate O3-I levels. In order to provide appropriate recommendations on diet and dietary needs, future studies are required to identify factors that influence O3-FA intake, including cost, availability, and dietary preferences. Healthcare professionals and those responsible for helping individuals achieve higher levels of O3-FA need to take into account these realistic challenges and offer solutions for closing the gap between dietary intake and cardio protective levels of O3-FA, including but not limited to supplementation, if appropriate.

## Figures and Tables

**Table 1:** Baseline Demographic Characteristics of Study Participants (*n* = 200, aged 18–80 years) by Country and Sex ^1^.

	United States				Germany			
	All ^2^ (*n* = 100)	Men (*n* = 46)	Women (*n* = 52)	*p*-Value	All (*n* = 100)	Men (*n* = 35)	Women (*n* = 65)	*p*-Value
Age in years, mean (SD) ^a^	29 (10)	28 (8)	31 (12)	0.225	40 (14)	41 (14)	40 (15)	0.722
Education% (*n*) ^b^								
High school and above	100 (100)	100 (46)	100 (52)	0.354	94 (93)	97 (33)	92 (60)	0.347
Frequency of Exercise, % (*n*) ^b^								
1–2 days a week	28 (28)	30 (14)	27 (14)	0.367	67 (49)	68 (19)	67 (30)	0.358
3–4 days a week	51 (50)	41 (19)	58 (30)		29 (21)	32 (9)	27 (12)	
Everyday	21 (21)	29 (13)	15 (8)		4 (3)	-	6 (3)	
Tobacco Use% (*n*) ^b^								
Never	82 (82)	74 (34)	90 (47)	0.191	54 (54)	60 (21)	51 (33)	0.538
Former	14 (14)	17 (8)	10 (5)		20 (20)	14 (5)	23 (15)	
Current	3 (3)	7 (3)	0		26 (26)	26 (9)	26 (17)	
Use of alcohol, % (*n*) ^b^								
Every day	2 (2)	0	4 (2)	0.244	5 (5)	6 (2)	5 (3)	0.459
Some days	85 (85)	91 (42)	81 (42)		84 (84)	88 (31)	81 (53)	
Never	13 (13)	9 (4)	15 (8)		11 (11)	6 (2)	14 (9)	
Body Mass Index, kg/m^2^, Mean (SD) ^a^	25 (5)	26 (4)	24 (5)	0.040	24 (4)	25 (3)	24 (3)	0.036

^1^ Comparison by sex-ANOVA (Analysis of Variance) was performed for comparing continuous variables ^a^ and chi-square tests ^b^ was performed for categorical variables (*p* < 0.05); ^2^ Two participants refused to provide their gender identity.

**Table 2:** Mean (95%CL) Omega-3 Index by sex and perception of dietary adequacy among adults in the United States and Germany (*n* = 200; ages 18–80 years) ^1^.

	**United States**						
By Sex ^2^				Do you think you get enough amounts of O3 from your diet?			
All (*n* = 100)	Men (*n* = 46)	Women (*n* = 52)	*p*-value	Yes (*n* = 30)	No (*n* = 41)	Unsure (*n* = 29)	*p*-value
4.3 (4.1–4.5)	3.8 (3.6–4.1)	4.8 (4.5–5.0)	<0.0001	4.4 (4.0–4.8)	4.1 (3.8–4.4)	4.5 (4.3–4.8)	0.167
	**Germany**						
By Sex				Do you think you get enough amounts of O3 from your diet?			
All (*n* = 100)	Men (*n* = 35)	Women (*n* = 65)	*p*-value	Yes (*n* = 24)	No (*n* = 40)	Unsure (*n* = 34)	*p*-value
5.5 (5.2–5.7)	5.3 (4.8–5.8)	5.6 (5.3–5.9)	0.369	5.7 (5.2–6.2)	5.2 (4.8–5.6)	5.6 (5.2–6.1)	0.322

^1^ ANOVA was performed for comparing continuous variables and chi-square tests were performed for categorical variables (*p* < 0.05); ^2^ Two participants refused to provide their gender identity.

## Supplementary Materials

The following are available online at www.mdpi.com/2072-6643/9/9/930/s1, Supplemental Table S1: Proportion (%) of adults reporting on the importance of improving or maintaining various dimensions of health in the United States and Germany; Supplemental Figure S1: Proportion of participants identifying food sources of omega-3 fatty acids in the United States (Panel A) and Germany (Panel B).
