# Supplementary material for: Discrepancy between Knowledge and Perceptions of Dietary Omega-3 Fatty Acid Intake Compared with the Omega-3 Index
Source: Nutrients. 2017 Aug 24;9(9):930. doi: 10.3390/nu9090930 (PMC5622690; doi:10.3390/nu9090930)
Supplement: Supplementary file 1 [file nutrients-09-00930-s001.zip › nutrients-218035-supplementary.pdf]

Supplemental Table S1. Proportion (%) of adults reporting on the importance of improving or maintaining various dimensions of health in the United States and Germany [1]

	Not important, n (%)	Somewhat important n (%)	Important n (%)	Very important n (%)
Health area	**United States**			
Overall wellness	-	5 (5)	20 (20)	75 (75)
Brain Health	-	3 (3)	21 (21)	76 (76)
Heart health	-	3 (3)	27 (27)	70 (70)
Immunity	-	13 (13)	31 (31)	56 (56)
Mood	-	13 (13)	34 (34)	53 (53)
Vision	-	14 (14)	36 (36)	48 (48)
Digestion	1 (1)	17 (17)	39 (39)	43 (43)
Bone health	2 (2)	13 (13)	44 (44)	41 (41)
Alzheimer's	8 (8)	19 (19)	26 (26)	34 (34)
	**Germany**			
Health area				
Overall wellness	4 (4)	7 (7)	33 (33)	54 (54)
Brain Health	4 (4)	8 (8)	35 (35)	48 (48)
Heart health	2 (2)	6 (6)	34 (34)	55 (55)
Immunity	3 (3)	8 (8)	29 (29)	58 (58)
Mood	5 (5)	19 (19)	31 (31)	42 (42)
Vision	7 (7)	19 (19)	41 (41)	25 (25)
Digestion	7 (7)	6 (6)	41 (41)	42 (42)
Bone health	-	8 (8)	47 (47)	41 (41)
Alzheimer's	6 (6)	14 (14)	46 (46)	21 (21)

[1]Percentages may not add up to 100, since data for categories 'Refused' and 'Don't know' are not shown

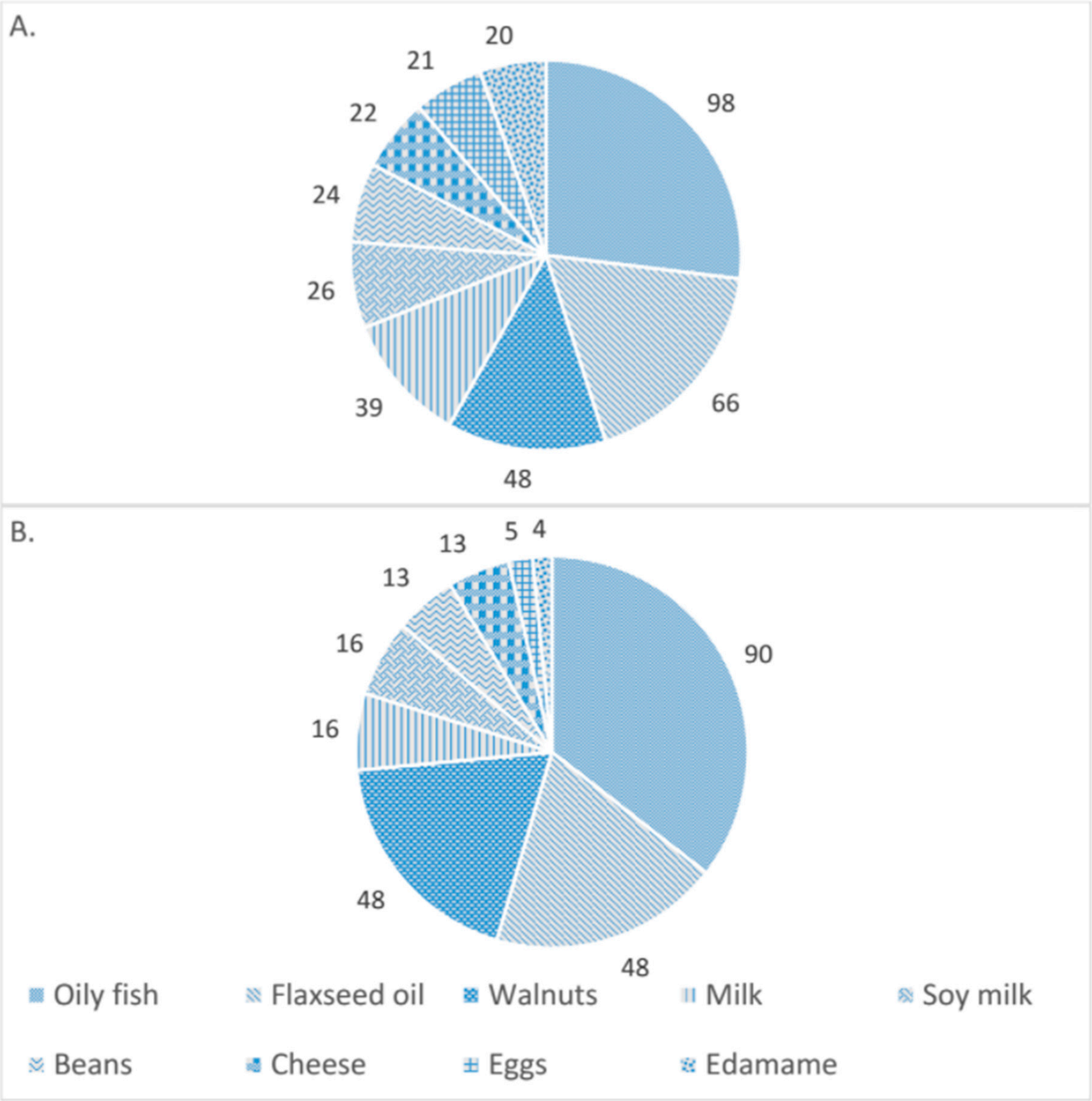

Supplemental Figure S1. Proportion of participants identifying food sources of omega-3 fatty acids in the United States (Panel A) and Germany (Panel B).
